# Classifying breast cancer and fibroadenoma tissue biopsies from paraffined stain-free slides by fractal biomarkers in Fourier Ptychographic Microscopy

**DOI:** 10.1016/j.csbj.2024.03.019

**Published:** 2024-03-24

**Authors:** Vittorio Bianco, Marika Valentino, Daniele Pirone, Lisa Miccio, Pasquale Memmolo, Valentina Brancato, Luigi Coppola, Giovanni Smaldone, Massimiliano D’Aiuto, Gennaro Mossetti, Marco Salvatore, Pietro Ferraro

**Affiliations:** a CNR-ISASI, Institute of Applied Sciences and Intelligent Systems “E. Caianiello”, Via Campi Flegrei 34, 80078 Pozzuoli, Napoli, Italy; b DIETI, Department of Electrical Engineering and Information Technologies, University of Naples “Federico II”, via Claudio 21, 80125 Napoli, Italy; c IRCCS SYNLAB SDN, Via E. Gianturco 113, Napoli 80143, Italy; d Clinica Villa Fiorita, Via Filippo Saporito 24, 81031 Aversa, Caserta, Italy; e Pathological Anatomy Service, Casa di Cura Maria Rosaria, Via Colle San Bartolomeo 50, 80045 Pompei, Napoli, Italy

**Keywords:** Quantitative Phase Imaging, Fourier Ptychographic Microscopy, Stain-free tissue analysis, Computational pathology, Fractal geometry, Machine learning

## Abstract

Breast cancer is one of the most spread and monitored pathologies in high-income countries. After breast biopsy, histological tissue is stored in paraffin, sectioned and mounted. Conventional inspection of tissue slides under benchtop light microscopes involves paraffin removal and staining, typically with H&E. Then, expert pathologists are called to judge the stained slides. However, paraffin removal and staining are operator-dependent, time and resources consuming processes that can generate ambiguities due to non-uniform staining. Here we propose a novel method that can work directly on paraffined stain-free slides. We use Fourier Ptychography as a quantitative phase-contrast microscopy method, which allows accessing a very wide field of view (i.e., mm^2^) in one single image while guaranteeing high lateral resolution (i.e., 0.5 µm). This imaging method is multi-scale, since it enables looking at the big picture, i.e. the complex tissue structure and connections, with the possibility to zoom-in up to the single-cell level. To handle this informative image content, we introduce elements of fractal geometry as multi-scale analysis method. We show the effectiveness of fractal features in describing and classifying fibroadenoma and breast cancer tissue slides from ten patients with very high accuracy. We reach 94.0 ± 4.2% test accuracy in classifying single images. Above all, we show that combining the decisions of the single images, each patient’s slide can be classified with no error. Besides, fractal geometry returns a guide map to help pathologist to judge the different tissue portions based on the likelihood these can be associated to a breast cancer or fibroadenoma biomarker. The proposed automatic method could significantly simplify the steps of tissue analysis and make it independent from the sample preparation, the skills of the lab operator and the pathologist.

## Highlights

• Breast cancer and fibroadenoma tissue slides are collected from patients.
• Fourier Ptychographic Microscopy (FPM) is employed as quantitative imaging.
• FPM phase-contrast maps of stain-free and paraffined tissue slides are imaged.
• Fractal features are used to characterize and classify multi-scale FPM images.
• Computational heatmaps of fractal lacunarity agree with the pathologist’s analysis.

## Graphical Abstract

**Figure:**
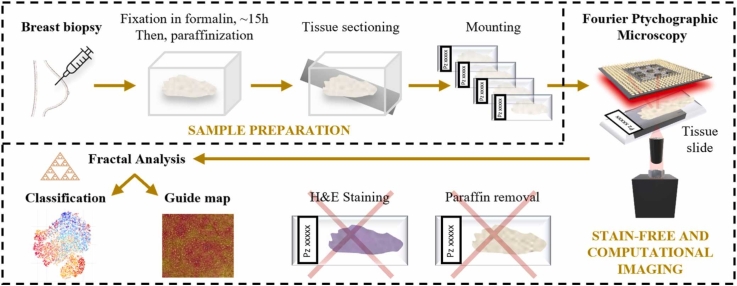

## 1 Introduction

Breast cancer represents one of the most monitored pathologies for women due to its high mortality and morbidity rate. In fact, the five-year survival rate in metastatic breast cancer is less than 30%. Recent data produced by the IARC (International Agency for Research on Cancer) report that in 185 examined countries, 2.3 million new cases (11.7%) of breast cancer were found with a mortality rate of 6.9% [1]. Also, the incidence of breast cancer is more common in high-income countries (571/100,000) than in low-income countries (95/100,000). Breast cancer encompasses a group of diseases characterized by different biological subtypes, with a molecular profile and specific clinical-pathological characteristics [2]. The diagnosis of breast cancer is based on clinical examination combined with imaging and confirmed by pathological assessment. The comprehensive pathological assessment of breast cancer should be performed in alignment with the World Health Organization (WHO) classification [3] and the eighth edition of the American Joint Committee on Cancer (AJCC) Tumour, Node, Metastasis (TNM) staging system [4], and includes not only anatomical considerations but also crucial prognostic insights tied to tumor biology, such as tumor grade, estrogen receptor (ER), progesterone receptor (PgR), human epidermal growth factor receptor 2 (HER2), and available gene expression data [5]. However, distinguishing breast cancer lesions from benign-looking subtypes like tubular or lobular carcinomas can be challenging. Fibroadenoma, a common benign breast tumor, often shares characteristics with breast cancer, complicating accurate diagnosis. Fibroadenomas are characterized by a proliferation of epithelial and stromal components, typically well-defined and distinct from surrounding breast tissue [6]. These benign growths exhibit a mixture of glandular and connective components. Given their similarity, current diagnostic techniques may struggle to differentiate between breast cancer and fibroadenoma [7].

In clinical practice, the pathological assessment of breast tissue is usually performed through needle aspiration, biopsy, or surgical excision. Immunohistochemical investigation such as the classical hematoxylin and eosin (H&E), the staining with specific antibody and other useful molecular tests are used for the characterization of breast cancer. However, the diagnosis results do not always coincide as they depend on several factors such as previous experience of the pathologist and sample preparation. Currently, accuracy of diagnosis is limited to 75% [8]. Rapid, automatic and less operator-dependent methods for the breast cancer diagnosis are still far from the actual needs. One approach to assist visual inspection is to employ simple methods to develop classifiers that analyze texture and morphological features computationally from standard microscope images of stained tissue slides. This would have the benefit to make steps forward in classifying benign and malignant tumors by automatic process and try to overcome the subjectivity in image analysis [9], [10]. Recently, interesting developments have been introduced in the histopathology field by digital pathology. The widespread use of slide scanning systems is mainly associated with the reduction of the costs of the scanning technology and digital storage. Whole Slide Imaging (WSI) has recently opened the route to several new possibilities [11], [12]. WSI allows very fast and high-resolution acquisition of entire tissue slides thus making available images of the biopsies in digital format with typical times compatible with clinical practice [13]. Accessing such a huge amount of data has favoured the use of data driven Artificial Intelligence (AI) for classification, diagnostics, and image enhancement to support researchers and pathologists in the accurate analysis of a patient’s slide [14], [15], [16], [17].

**Fig. 1:**
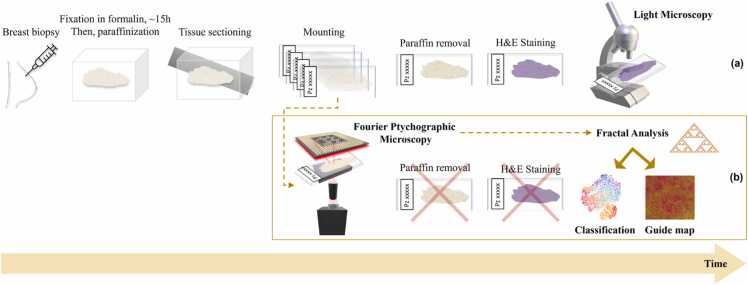
Sketch of the process of tissue slide analysis. (a) Conventional light microscopy analysis. (b) Proposed stain-free method.

In accordance with current protocols, pathologists typically assess prepared and stained slides based on their familiarity with the typical appearance of healthy tissue morphology as revealed by the stain's intrinsic filter. However, various factors can substantially influence the quality of immunohistochemistry and the overall tissue preparation process. These factors include storage duration, oxidation, hydrolysis, tissue processing duration, fixation method, and fixation duration. Fixation in 10% neutral buffered formalin, for approximately 15 h, and slide storage in paraffin is of great importance to preserve the tissue samples. Usually, coating or dipping coating with paraffin is provided in order to embed and seal the tissue slides to reduce oxidation. Paraffin must be removed in an incubator, then the slide must be stained, as sketched in Fig. 1(a) [18]. Staining is a process characterized by a large failure rate. Uneven staining [19] can occur during the process of paraffin removal, or due to incorrect sectioning, overly dehydrated tissue, poorly infiltrated tissue, or water provoking under-staining of cytoplasmic structures [20]. Also, the use of formalin can cause over-drying and searing of the outer edges of the tissue when the slide is excessively exposed to sunlight, thus provoking an incorrect appearance and loss of the nuclear details [20]. Microscopy observation of the slides under a Light Microscope (LM) returns false colour images showing the stained areas with higher contrast, while the tissue inner structures that do not bind to the stain are returned with poor contrast. While this observation method is widely employed in clinical practice, its inherent ambiguity can result in misinterpretations. This ambiguity stems from operator and lab dependencies, as well as the absence of an absolute reference for comparing images, especially when pixel values cannot be directly linked to a physical measure [8]. Similarly, algorithms for automatic image analysis and even deep learning architectures can be affected by such stain-induced ambiguities [21].

With the aim to avoid the ambiguities associated with staining, label-free methods have emerged. Raman spectroscopy can provide sample information about biomolecular alterations in non-destructive way and label-free mode [22]. Furthermore, Raman spectroscopy could be combined with machine learning (ML) to automatically perform the spectral analysis. High-definition Fourier Transform Infrared (FT-IR) imaging has been used to find a spectroscopic signature for breast cancer classification [23]. Non-linear optical imaging has been also employed for the in vivo tissue histology [24]. Among these several label-free techniques, Quantitative Phase Imaging (QPI) emerged as a class of methods to image label/stain-free biological samples while providing the necessary contrast for downstream analysis, physiology and histopathology observations [25]. QPI methods measure the optical path delay introduced by the biological sample on the light probe, which is linked to biophysical quantities. In QPI, the optical readout is the phase-contrast map. Unlike LM images of stained specimens, each pixel of a phase-contrast map is proportional to the optical thickness, i.e. the product between the physical thickness and the integral along the optical axis direction of the refractive index. Similarly, the dry mass can be measured. Both quantities are contingent upon the local density of the specimen [26]. This mechanism provides the contrast needed for analyzing stain-free tissue slides without ambiguities. Different QPI approaches have been proposed to inspect biological tissue slides in stain-free mode, including Digital Holography (DH) [27], [28], [29], Fourier Ptychographic Microscopy (FPM) [30], [31], [32], [33], [34], [35], [36], micro-optical coherence tomography [37], SLIM [38], and the above-mentioned WSI [11], [12]. Among the different QPI approaches, FPM is preferred to favourably stretch the optical constraint that limits the obtainable space bandwidth product. In FPM, phase-contrast imaging over a wide Field of View (FoV) is accessible by selecting an optical configuration typically adopting low magnification microscope objectives (MOs). For most of the benchtop microscopes, this choice means sacrificing the available lateral resolution. In FPM, angle diversity is introduced in the illumination pattern with the aim to enhance the resolution according to a synthetic aperture principle [30], [31], [32], [33], [34], [35], [36]. A set of bright-field and dark-field images is captured, each one transferring a subset of spatial frequencies of the sample. In particular, the large angle light probes (dark field images) have the effect of conveying the high frequency details into the MO Numerical Aperture (NA), thus transferring them within the system bandpass cutoff. Then, a phase-retrieval process estimates the high-resolution complex amplitude from the set of low-resolution intensities. The effect is a mm^2^-cm^2^ size FoV image with submicron lateral resolution. This is ideal to investigate histopathology slides where small tissue portions are not necessarily representative of the condition of the patient undergoing biopsy and the inspection of the entire slide is necessary. FPM has been used with various coded illumination schemes [39] for imaging cell cultures and tissue slides [30], [40] in applications ranging from biology research and drug testing to mechanobiology [30], [34], [41]. Recently, deep learning methods have been employed to fasten the reconstruction process [36], [42], [43] and to make FPM microscopes more robust against misalignments, thus helping the ongoing process of translating FPM to clinical practice [35], [44].

Here we consider classification between breast cancer and fibroadenoma tissue slides as a clinical case to test the potentiality of FPM imaging combined with fractal geometry and machine learning as label-free, rapid, automatic and less operator-dependent diagnostic tool. In particular, we use FPM to image and analyse breast tissue slides from ten patients in stain-free modality. We accurately identify the tissue portions exhibiting breast cancer from the fibroadenoma areas. ML is applied by extracting meaningful features from the wrapped (i.e., modulus 2π) FPM phase-contrast maps. The features are used to train a classifier to infer the class each image patch belongs to. Then, by using a max-voting approach specifically developed for digital histopathology, the proposed method is able to provide accurate classification at the single patch level, image level, and patient’s level with increasing minimization of the classification error (i.e., on average, 15.2%, 6.0%, and 0.0%, respectively), that is the percentage of misclassification errors with respect to the total number of classified elements. As a result of this analysis, we provide a very accurate overall classification of the patients’ slide to furnish a first automatic indication to the pathologist. Besides, we create a heatmap of the most relevant parameter for classification, which can serve as a guide to establish the areas where the breast cancer phenotype is more or less expressed.

The stain-free process we propose is sketched in Fig. 1(b). The idea is to image the stain-free slides without removing the paraffin. Paraffin acts preserving the tissue slides. However, it can be detrimental for FPM phase imaging. Indeed, paraffin can act as a pure-phase layer that introduces an additional optical path delay and provokes severe phase wrapping. Nevertheless, we show that paraffin does not affect the proposed analysis of FPM images. Essentially, the phase wrapping pattern obtained from FPM can be used as a fingerprint to characterize and classify the different portions of the image. In particular, we rely on a recently developed analysis framework based on elements of fractal geometry. Fractal geometry is a branch of math particularly suitable to describe natural objects and their complexity [45], [46]. In microscopy, it has been applied to various problems, e.g. to describe the capillary system in angiography [47], the structure of neuron networks in LM images of brain tissue slides [48], to phenotype tumour cells [49], and in scattering-based cytometry to characterize the complexity of scattering patterns of single cells [50] and their link to the intracellular composition and distribution of organelles, e.g. the mitochondrial network in healthy and precancerous epithelial cells [51]. Recently, we applied the fractal analysis to wrapped holographic phase-contrast maps of marine microalgae and microplastics to define a fingerprint of microplastic items and identify them in water samples [52]. Indeed, complexity descriptors like fractal dimension and lacunarity [47], [53], [54] are particularly useful to characterize the distribution of the phase jumps within each single cell [52]. Here we apply such elements of fractal geometry to the FPM wrapped phase-contrast images, i.e. we describe the structure of phase-jumps (or “lacunes”) at the whole image level to train the classifier. Fractal descriptors are known to describe better than morphological and textural features the distribution of the lacunes, their connections within the structure of the object, statistical similarities, and the way these traits scale up and down to different sizes. This analysis is facilitated by the "multi-scale" capabilities inherent in both FPM imaging, which ensures a large space-bandwidth product, enabling zooming in and out without sacrificing spatial details, and fractal geometry, where descriptors are defined based on the box size used for measurement. For this reason, fractal features are expected to provide a distinctive characterization of the different biological frameworks of a breast fibroadenoma or a breast cancer tissue imaged by FPM. Hence, we show the effectiveness of fractal descriptors to classify the stain-free digital images of breast tissue biopsies recorded by FPM without removing paraffin, as sketched in Fig. 1(b).

## 2 Materials and methods

### 2.1 FPM complex amplitude estimate

FPM [30], [31] was initially proposed by G. Zheng et al. [55]. It estimates the object complex amplitude from a set of intensity captures relying on phase retrieval algorithms [33].

**Fig. 2:**
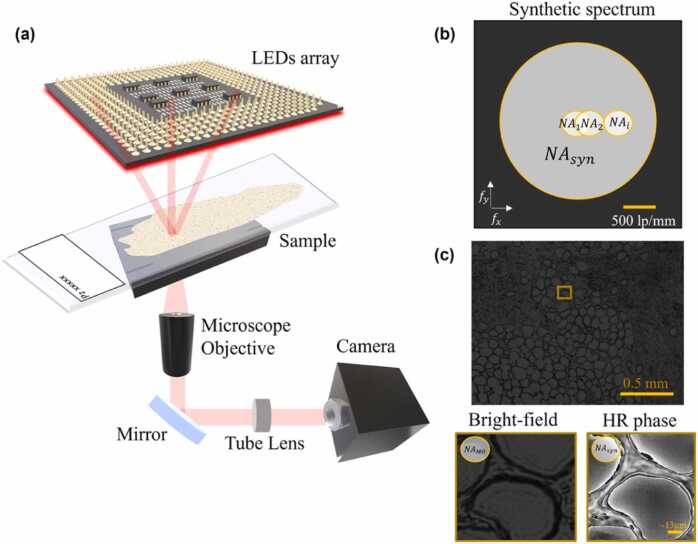
FPM acquisition scheme. (a) Experimental setup, where we intentionally enlarged the sketch of the sample tissue slide in the acquisition plane. (b) Sketch of the Fourier synthetic spectrum that shows the NA enhancement. (c) Top: example of bright-field image corresponding to the central LED. Bottom: zoom-in detail of the area marked by the yellow box. Bottom left: low resolution bright-field intensity. Bottom right: corresponding high resolution wrapped phase-contrast map.

The FPM system is built in order to follow synthetic aperture principle, by source-coding an array of 177 light sources that sequentially turn on and off to illuminate the specimen from different directions. In our configuration, the MO has low numerical aperture (NA) for ensuring the wide FoV, while the illumination source is a planar array of LEDs. The system setup and acquisition working principle are sketched in Fig. 2. Sequentially turning on each LED, the object on the sample plane is probed by different light sources with illumination angles that depend on their position in the source array. The central LEDs probe the object perpendicularly to the sample plane and generate bright-field intensity images on the camera, while the outermost ones provide a beam grazing the sample at a certain angle, generating dark-field intensity images on the camera. In the frequency domain, a light beam with high illumination angle shifts the illumination NA towards high frequencies.

If O(r) represents the object on the sample plane (*r* as spatial coordinate) and $e^{j2\pi \mathrm{fr}}$ the complex field emitted from a single LED (*f* as frequency), the transmitted complex field through the object is

(1) $$O^{\prime }\left(r\right)=O(r)e^{j2\pi \mathrm{fr}}\overset{f=-\frac{\sin(\theta )}{\lambda }}{\to }O(r)e^{-j2\pi \frac{\sin(\theta )}{\lambda }r}\overset{\sin(\theta )\approx \theta }{\to }O(r)e^{-j2\pi \frac{\theta }{\lambda }r}$$

where *θ* refers to the illumination angle and *λ* to the LED wavelength. In Eq. 1, the linear correlation between the illumination angle and the frequency highlights the influence of the angle variation on the frequency values. Hence, combining properly the LED NAs in the Fourier spectrum, a bigger NA can be synthetized covering a wide frequency range (Fig. 2(b)), i.e.

(2) $$\mathrm{SNA}={\mathrm{NA}}_{\mathrm{MO}}+{\mathrm{NA}}_{\mathrm{ill}}$$

where *SNA* is the synthetic numerical aperture, ${\mathrm{NA}}_{\mathrm{MO}}$ the NA of the MO, and ${\mathrm{NA}}_{\mathrm{ill}}=\frac{\lambda }{2\pi }k_{\max}\propto \sin\vartheta_{\max}$ the illumination NA obtained by the maximal spatial frequency of the illumination that corresponds to the illumination wave vector with the largest angle $\vartheta_{\max}$ (i.e., the outermost LED) [34], [56].

The central LEDs contribute to image the basic structure of the object (i.e. the low spatial frequency content), while the external LEDs provide the finest details (dark field images). The captured images (per each LED) show the spatial resolution corresponding to the 0.1 NA of the MO, and their intensities can be estimated as follows

(3) $$I_{\mathrm{LR},i}={\left|{\mathrm{FT}}^{-1}\left\{\tilde{O^{\prime }}\left(f-f_{i}\right)H(f)\right\}\right|}^{2}$$

where ${\mathrm{FT}}^{-1}$ is the inverse Fourier Transform (FT), $\tilde{O^{\prime }}$ the FT of $O^{\prime }\left(r\right)$ and H the FT of the system impulse response, i.e. the transfer function. An example of bright-field image of one of the breast cancer tissue slides, acquired by switching on the central LED, is reported in Fig. 2(c). In Fig. 2(c) we also show a zoom-in detail of the area marked by the yellow box. In particular, we show the enlarged detail of the low-resolution bright-field intensity and the corresponding high-resolution wrapped phase-contrast map.

The relationship between spatial domain (left side Eq. 3) and frequency domain (right side Eq. 3) is pivotal in the phase retrieval algorithm that is based on an iterative updating of the estimated complex amplitude between both domains until the convergence of the metric used is reached. After several iterations, the high-resolution complex amplitude is obtained, whose phase distribution is given by

(4) $$\varphi \left(I_{\mathrm{LR},0},f\right)=\mathrm{arctg}(\hat{O}\left(I_{\mathrm{LR},0},f\right))$$

where $I_{\mathrm{LR},0}$ is the initial guess of the iterative algorithm and $\hat{O}$ is the high-resolution complex field.

### 2.2 FPM experimental setup

The experimental apparatus for FPM is sketched in Fig. 2. Here, we use a × 4 plan achromatic MO (Plan N, 0.1 NA, Olympus) and a 32 × 32 RGB LEDs array, set at red wavelength (632 nm), with a bandwidth of ∼20 nm. The distance between LEDs on the illumination array is 4 mm. A 400 mm tube lens redirects the transmitted light beam in a charge coupled device (CCD) camera (Photometrics Evolve 512, 12-bit quantization), with 4.54 µm pixel pitch. The sample plane is 4.67 cm far from the illumination source.

An Arduino board guided by a main MATLAB® script guides the sequential illumination of 177 LEDs. The acquired images (low resolution) have a × 4.29 magnification and a size of 1460 × 1940. Here, the EPRY (Embedded Pupil function Recovery) phase retrieval algorithm is applied for FPM reconstructions [57], [58]. EPRY algorithm allows retrieving both the Fourier spectrum of the observed object and the pupil function of the imaging system used for the experiments. This simultaneous recovery process permits to avoid a priori knowledge on system errors, such as aberrations.

As in other microscopy systems, defocus can occur depending on the optical system depth of field (DOF). This is inversely proportional to the numerical aperture (NA), i.e. $\mathrm{DOF}∼\frac{\lambda n}{{\mathrm{NA}}^{2}}$. Here, we use NA=0.1 and λ=0.632 μm, hence the DOF∼63.2 μm. This is one of the advantages of using a small NA system to synthesize a larger NA optical apparatus.

Being the tissue slice thickness ∼ 4 µm, the probed object can be considered thin for our FPM system and thus the reconstruction algorithm works well. In case of thicker tissue slices, e.g. 15/20 µm, different light propagation models and FPM reconstruction algorithms should be used, such as the multi-slice model under the first Born or Rytov approximations [59].

To promote the convergence of the phase retrieval algorithm and the assumption of plane wave, the images are cropped in 100 × 100 pixels, obtaining 266 patches. The final image (high-resolution) reaches a size of 7000 × 9500 pixels (where each high-resolution patch is 500 ×500 pixels sized). The spatial resolution of our system is demonstrated to reach 0.5 µm over a ∼3 mm^2^ FoV area.

The entire FPM process for one image takes ∼32 min by using an Intel i7–4790 CPU running @ 3.60 GHz and 16 GB RAM. For each patch, 7.2 s are needed to complete 60 iterations to end the phase-retrieval FPM process.

### 2.3 Fractal analysis of wrapped FPM maps

**Fig. 3:**
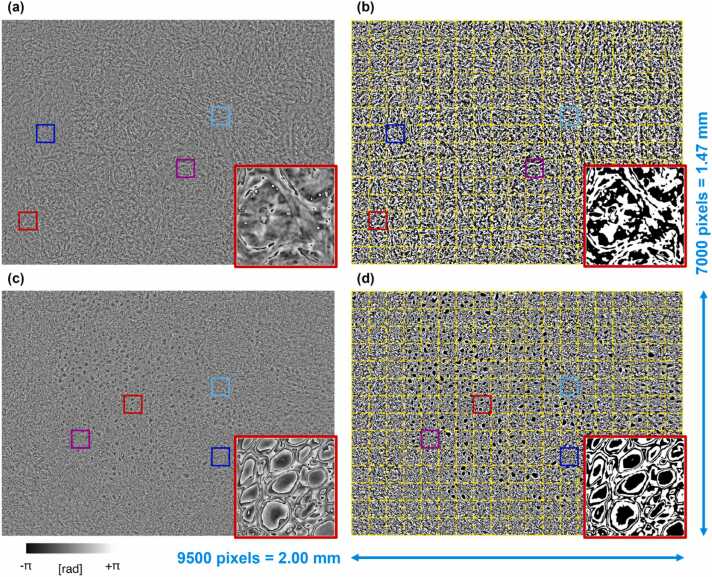
Examples of fibroadenoma (a,b) and breast cancer (c,d) FPM images. (a,c) Wrapped FPM maps. (b,d) Binary FPM maps obtained by zero-thresholding the wrapped FPM maps in (a,c), respectively, with overlapped in yellow the 14 × 19 patches (500 ×500 square pixels) dividing the overall 7000 × 9500 FoV. The other zoomed-in insets are displayed in Supplementary Figs. S1(a,b), corresponding to the fibroadenoma in (a,b) and to the breast cancer in (c,d), respectively.

An example of wrapped FPM maps related to a fibroadenoma tissue slide and a breast cancer tissue slide are displayed in Fig. 3(a,c), respectively. Due to the presence of paraffin inside the imaged tissue biopsies, a dense distribution of phase jumps characterizes the wrapped FPM maps. Nevertheless, differences between the underlying tissue structures can be inferred from the wrapped FPM maps, as highlighted in the red insets in Fig. 3(a,c). In order to quantitively characterize them, we measured an ad hoc feature set based on the fractal geometry theory. Fractal parameters are measured over a binary map consisting of full and empty areas. In previous works, we experimentally verified that using a zero threshold in maps that range between -π and π (i.e. a symmetric interval around the zero) allowed us to emphasize the discontinuities provoked by the jumps and to characterize the lacunes in the resulting binary masks [52]. Here we use a different QPI method with respect to Ref. [52], and we work at the whole image level rather than single cell level. However, we found convenient to apply the same criterion since again the wrapping interval is symmetric around the null value. The binary FPM maps are shown in Fig. 3(b,d) for the fibroadenoma and the cancer tissue slides, respectively. Then, the binary FPM maps, made of 7000 × 9500 square pixels, were divided into non-overlapping 14 × 19 binary patches made of 500 × 500 square pixels, as shown by the yellow grid in Fig. 3(b,d). Finally, for each of the 14 × 19 patches, the 13 fractal parameters defined in Ref. [52] and described in the Supplementary Section S1 were computed, namely the fractal dimension, lacunarity index, fill ratio, regularity index, vertex density, vertex lacunarity index, vertex regularity index, fractal dimension contrast, lacunarity contrast, vertex lacunarity contrast, fractal dimension RMSE, lacunarity RMSE, and vertex lacunarity RMSE. Furthermore, for each patch, other 2 features were added, which can be related to the fractal behaviour of the wrapped FPM maps [60], i.e. the standard deviation and the entropy, which were computed directly from the phase values. Indeed, standard deviation quantitatively measures the variation of phase values with respect to the phase mean value, thus it can be correlated to the frequency and the intensity of the phase jumps. In fact, more frequent and more intense phase jumps lead to higher standard deviations but, at the same time, they change the binary FPM map and the corresponding fractal features. Instead, entropy is computed from the histogram of phase values and is a statistical measure of the randomness of phase jumps within the FPM map.

### 2.4 Machine learning classification

To discriminate between fibroadenoma and breast cancer tissue biopsies using the 15 FPM fractal features, we trained several machine learning (ML) models using a 10-fold cross-validation approach through the Classification Learner App of MATLAB® R2023a. Specifically, we utilized the support vector machine (SVM), k-nearest neighbors (KNN), and linear discriminant analysis (LDA) algorithms [61]. For SVM, we employed the preset Linear SVM with a linear kernel, automatic kernel scale, a box constraint level of 1, and data standardization. For KNN, we utilized the preset Fine KNN with 1 as number of neighbor, Euclidean metric distance, equal distance weight, and data standardization. In all cases, no feature selection or model parameter optimization was performed for the ML models.

To evaluate the ML classification performance, we exploited as metrics the recall, precision, and accuracy. Given the classes A and B, let true positives (TP) be the number of correctly classified elements belonging to class A, let true negatives (TN) be the number of correctly classified elements belonging to class B, let false negatives (FN) be the number of elements belonging to class A but misclassified as class B, and let false positives (FP) the number of elements belonging to class B but misclassified as class A. The recall (Rec), precision (Prec), and accuracy (Acc) are defined as

(5) $$\mathrm{Rec}=\frac{\mathrm{TP}}{\mathrm{TP}+\mathrm{FN}},$$

(6) $$\mathrm{Prec}=\frac{\mathrm{TP}}{\mathrm{TP}+\mathrm{FP}},$$

(7) $$\mathrm{Acc}=\frac{\mathrm{TP}+\mathrm{TN}}{\mathrm{TP}+\mathrm{TN}+\mathrm{FP}+\mathrm{FN}}.\;$$

Hence, accuracy represents the probability of correctly classifying a general element. Recall quantifies the probability of correctly classifying an element that truly belongs to a specific class, while precision measures the probability of correctly identifying an element as belonging to a specific class among all the elements classified as such. Instead, another common classification metric is related to the receiver operating characteristic (ROC) curve, i.e., the area under the ROC curve (AUC) [62]. The ROC curve illustrates the true positive rate (TPR) vs. the false positive rate (FPR) at different threshold values of the trained ML model. TPR corresponds to the recall, while FPR is defined as

(8) $$\mathrm{FPR}=\frac{\mathrm{FP}}{\mathrm{TN}+\mathrm{FP}}$$

and is the probability that a true positive is missed during classification.

## 3 Results

### 3.1 ML classification of breast tissue slides

**Fig. 4:**
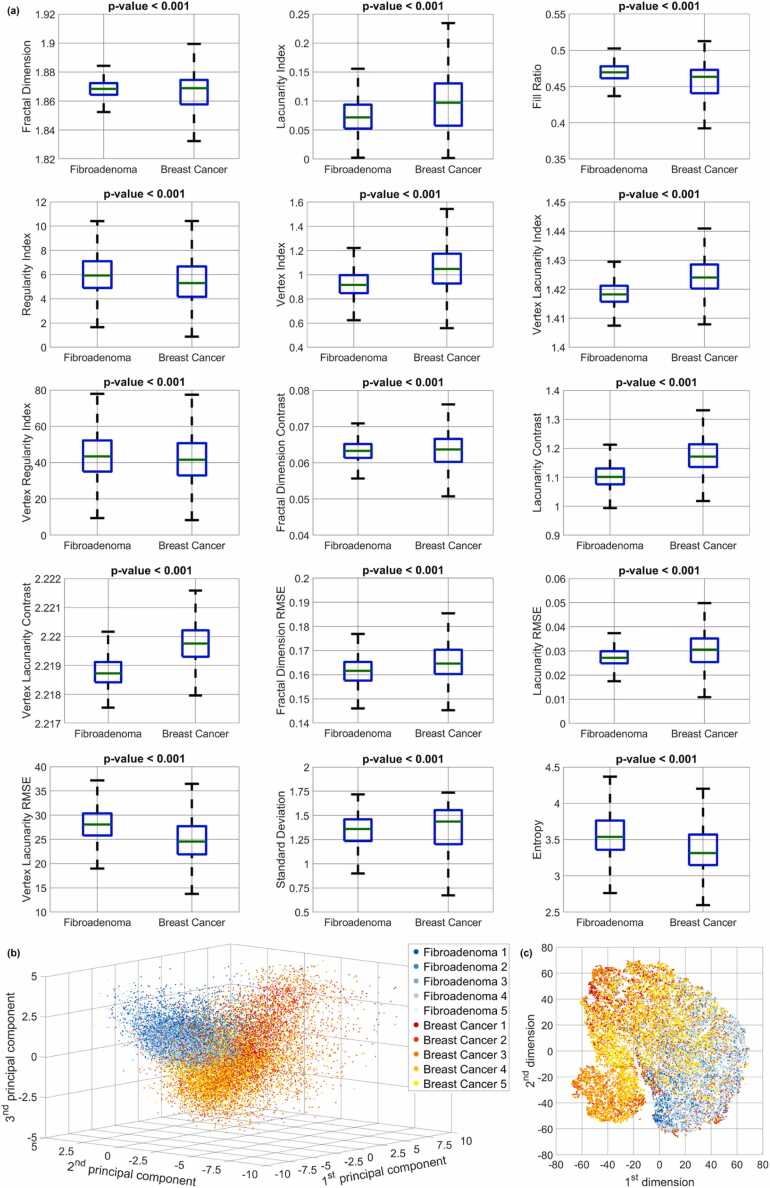
Inspection of the overall dataset by separating the contributions of the 10 tissue biopsies (5 fibroadenoma and 5 breast cancer tissue biopsies). (a) Boxplots of the 15 fractal features used for classification. (b) PCA scatter plot. (c) t-SNE scatter plot.

The FPM experimental setup described in Material and Methods has been employed to image tissue biopsies taken from 10 patients, i.e. 5 patients with fibroadenoma and 5 patients with breast cancer. For each patient (i.e., for each tissue biopsy), the wrapped FPM maps of 13 different FoVs have been recorded, like those displayed in Fig. 3(a,c). In the corresponding insets, phase jumps related to the presence of paraffin can be observed. In fact, the paraffin layer acts as a pure phase object on the image that adds an unknown phase contribution to the phase delay introduced by the tissue. Thus, it is very likely that unpredictable phase jumps appear on the image although the tissue, per se, would not provoke this random wrapping. In image portions containing the tissue, it is not possible to decouple the two contributions (since FPM in transmission is an integral imaging mode that cannot resolve the phase contributions along the optical axis). Other zoomed-in insets with these artefacts are displayed in Supplementary Figs. S1(a,b). Instead, at the tissue boundaries, we noticed areas where the paraffin layer is present alone without the tissue covering it. In those small areas, the phase delay introduced by the sole paraffin can be observed, as shown in the example of Supplementary Fig. S1(c). Hence, we exploited fractal geometry to describe the information encoded in the phase jumps generated by the paraffin layer overlapped to the breast tissue slide. According to the fractal analysis described in Material and Methods, each FoV has been divided into 266 non-overlapped patches, according to the grid sketched in Fig. 3(b,d). Finally, for each patch, 15 fractal parameters have been measured. In summary, the collected dataset is made of 15 fractal features related to the overall 34,580 FPM patches, which are taken from 130 wrapped FPM maps belonging to the tissue biopsies of 10 patients (5 fibroadenoma patients and 5 breast cancer patients). The boxplots related to the 15 fractal features used for classification are displayed in Fig. 4(a). For all of them, the p-value computed by the two-sample Student’s t-test [63] is lower than 0.001, meaning that there is statistical significance in the observed differences in terms of FPM-based fractal features between the fibroadenoma and breast cancer tissue biopsies.

To inspect the collected dataset in terms of fractal features, the principal component analysis (PCA) has been implemented to reduce its dimensionality [64]. Fig. 4(b) displays the first three principal components, revealing distinct clusters formed by the 17,290 fibroadenoma patches and the 17,290 breast cancer patches. These clusters are notably well-defined and separated from each other. Furthermore, in order to perform a more detailed data inspection within the two clusters, the t-distributed stochastic neighbor embedding (t-SNE) algorithm has been exploited [65]. The results of the t-SNE analysis are depicted in Fig. 4(c), where the 17,290 patches from the five fibroadenoma tissue biopsies (i.e., patients) form a cohesive cluster represented by blue points. Conversely, the 17,290 patches from the five breast cancer tissue biopsies (i.e., patients) are divided into two interconnected clusters, as indicated by the red points. This means that the paraffined breast cancer patches, when characterized by a fractal feature set, exhibit a greater intra-class variability with respect to the fibroadenoma patches. This is reasonable considering that the breast cancer phenotype is not homogeneously expressed over the entire FoV, rather it is localized in certain image areas. Besides, there are patches belonging to breast cancer patients that do not exhibit that phenotype and cluster in a separate region of the t-SNE diagram. Nevertheless, as well as the PCA analysis, also the t-SNE analysis confirms the good inter-class separation. It is important to highlight that neither the PCA results nor the t-SNE results were utilized for classification purposes. Instead, they were solely employed to reduce the dimensionality of the feature set, facilitating visual inspection of the dataset characterized by the 15 fractal features.

**Fig. 5:**
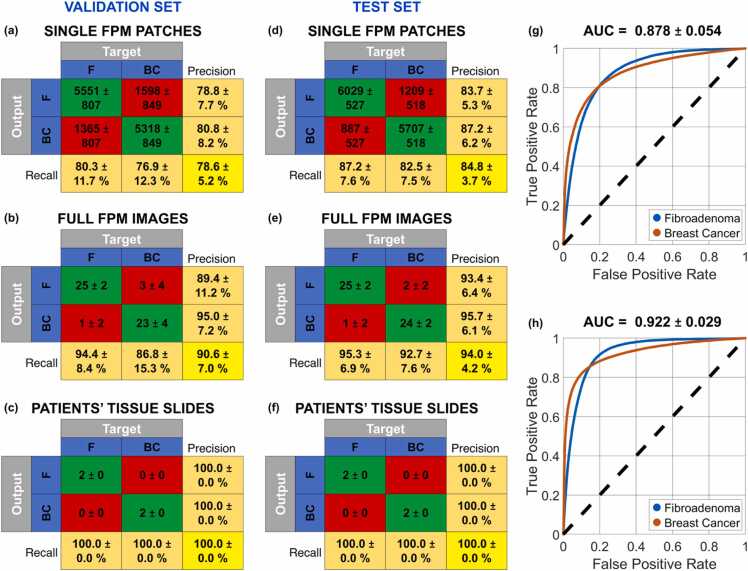
Classification between fibroadenoma (F) and breast cancer (BC) patients based on an SVM model. (a,d) Average and standard deviation values of the nine confusion matrices obtained over the validation and test sets, respectively, made of 13,832 FPM patches belonging to 52 FPM images of 4 tissue biopsies. (b,e) Average and standard deviation values of the nine confusion matrices obtained over the validation and test sets, respectively, made of 52 FPM images belonging to the tissue biopsies of 4 patients, obtained after max-voting of the FPM patch classes. (c,f) Average and standard deviation values of the nine confusion matrices obtained over the validation and test sets, respectively, made of 4 tissue biopsies belonging to 4 patients, obtained after max-voting of the FPM image classes. (g,h) Average ROC curves of the nine classification problems related to the FPM patches of the validation and test sets, respectively, with the corresponding AUC values reported at the top. The employed metrics are defined in Section 2.4.

The data inspection performed in Fig. 4 suggests that the fractal feature set could be suitable to solve a classification problem for detecting a breast cancer tissue biopsy in respect to a fibroadenoma one. Moreover, the substantial separation between the two classes suggests that a small training set could be enough to expect a good generalization at the inference step. For this reason, the training set has been considered in the worst possible condition, i.e. by using all the FPM patches of just 2 tissue biopsies, that are one fibroadenoma and one breast cancer patient. A validation set of 4 tissue biopsies (i.e., two fibroadenoma and two breast cancer patients) and a test set of 4 tissue biopsies (i.e., two fibroadenoma and two breast cancer patients) have been considered (see Table 1). To avoid any bias that could be induced by a favorable selection of the training set, all the nine possible splits among the 6 patients of the training and validation sets have been considered, while keeping fixed the test set. Hence, for each split, the training set is made of the 6,916 FPM patches belonging to 2 patients (one fibroadenoma and one breast cancer patient) and the validation set is made of the 13,832 FPM patches belonging to the remaining 4 patients (two fibroadenoma and two breast cancer patients), as summarized in Table 1. For each of these nine classification problems, some ML models have been trained (see Section 2.4), i.e., the SVM, the KNN, and the LDA [61]. Then, for each of the nine classification problems, we selected the ML model providing the best classification accuracy over the corresponding validation set in terms of patients’ tissue slides. The average and standard deviation values about the resulting nine confusion matrices related to the 13,832 FPM patches of the validations sets are summarized in Fig. 5(a). A 78.6 ± 5.2% accuracy is reached, which is a satisfactory result considering that the training set is made of just 2 patients. The corresponding average ROC curve is shown in Fig. 5(g), in which a 0.878 ± 0.054 AUC is obtained.

**Table 1:** Splitting the classification dataset between fibroadenoma and breast cancer tissue biopsies.

		*Fibroadenoma*	*Breast Cancer*
*Training Set*	# FPM patches	3,458	3,458
	# FPM images	13	13
	# Patients	1	1
*Validation Set*	# FPM patches	6,916	6,916
	# FPM images	26	26
	# Patients	2	2
*Test Set*	# FPM patches	6,916	6,916
	# FPM images	26	26
	# Patients	2	2

As discussed before, each imaged FoV is made of 266 non-overlapped FPM patches. Thus, for each FoV, 266 possible classes are predicted by the ML classifier. In order to predict the class related to a specific FoV, a max-voting strategy can be applied [66], i.e. the class the imaged FOV belongs to is represented by the mode of the corresponding 266 patch classes. In this way, exploiting the intrinsic correlation between patches that belong to the same imaged FoV, the accuracy in classifying the overall FoV instead of the single patches raises up to a remarkable 90.6 ± 7.0% within the validation set made of 52 elements (see the average ± standard deviation confusion matrix in Fig. 5(b)).

In turn, 13 FoVs are imaged for each patient. Therefore, max-voting can be further exploited to combine the predicted classes of all the 13 FoVs belonging to the same slide in order to predict whether the corresponding tissue biopsy is a fibroadenoma or a breast cancer. As highlighted in Fig. 5(c), a remarkable 100.0 ± 0.0% accuracy is reached in this classification task within the validation set made of 4 patients.

The SVM model provided a 100% classification accuracy over the 4 patients of the validation set in all the nine classification problems. Therefore, it has been always selected as the best ML model, thus obtaining the average ± standard deviation confusion matrices in Fig. 5(a-c). Instead, the KNN and the LDA have provided a 100% classification accuracy over the 4 patients of the validation set only in seven and six of the nine classification problems, respectively.

Finally, each of the nine SVM trained models has been employed to classify the other 4 patients of the test set. According to the average ± standard deviation confusion matrices displayed in Fig. 5(d-f), an 84.8 ± 3.7% accuracy is obtained with the 13,832 test FPM patches (with a 0.922 ± 0.029 AUC corresponding to the ROC curve in Fig. 5(h)), a 94.0 ± 4.2% accuracy is obtained with the corresponding 52 test FPM images, and a remarkable 100.0 ± 0.0% accuracy is obtained with the corresponding 4 patients. Hence, regardless the selection of the training set and despite its small size, the maximum classification accuracy can be reached over an independent test set never “seen” by the classifier.

It is worth noting that the max-voting strategy is based on the data redundancy due to both the intrinsic large space bandwidth product of FPM (i.e., the large number of patches) and the acquisition of multiple FPM FoVs of the same breast tissue slide. Max-voting has to be applied with some caution though, since its effectiveness strictly depends on the classification accuracy at the single-patch level. Indeed, an excessive number of misclassifications at the patch level could potentially propagate to higher levels, affecting both the classification performance about FPM images and the overall assessment at the patient level. However, the effective combination between FPM and fractal geometry allows obtaining high classification performance at the single-patch level, which are then further amplified by the max-voting strategy, as summarized by the confusion matrices in Fig. 5(d-f).

### 3.2 Fractal FPM heat maps as a guide for pathologists

Fractal geometry offers an alternative perspective for describing natural objects compared to traditional Euclidean geometry. The powerfulness of fractal geometry lies in its ability of describing patterns that are intrinsic to a certain object, thus accessing its inner complex nature. In this way, a more distinctive characterization can be extracted from the analyzed phenomenon. This is possible since fractal geometry involves a multi-scale analysis of the imaged object, i.e. it tries to describe and quantify the replication of specific patterns at different scales within the same object. Hence, the multi-scale analysis offered by fractal geometry complements the multi-scale imaging capabilities of FPM, contributing to the very good classification performance observed thus far. In addition to the sole classification, we provide an additional source of information that could be meaningful as a guide for more in-depth studies by pathologists, i.e. fractal heat maps. Among the several fractal features, the lacunarity index has been often exploited due to its higher correlation with biological phenomena [45]. For example, lacunarity has been employed in the magnetic resonance imaging for distinguishing benign and malignant breast cancer [67] or for differentiating the grades of glioma [68]. It has been also used in other microscopy imaging techniques as prognostic indicator of clinical outcome in early breast cancer [69], for the diagnosis [70] and the identification of the severity level of prostate cancer [71], [72], or for the detection of the Alzheimer’s disease [73]. Actually, the lacunarity index measures the distribution of the hole sizes within a certain structure [54].

**Fig. 6:**
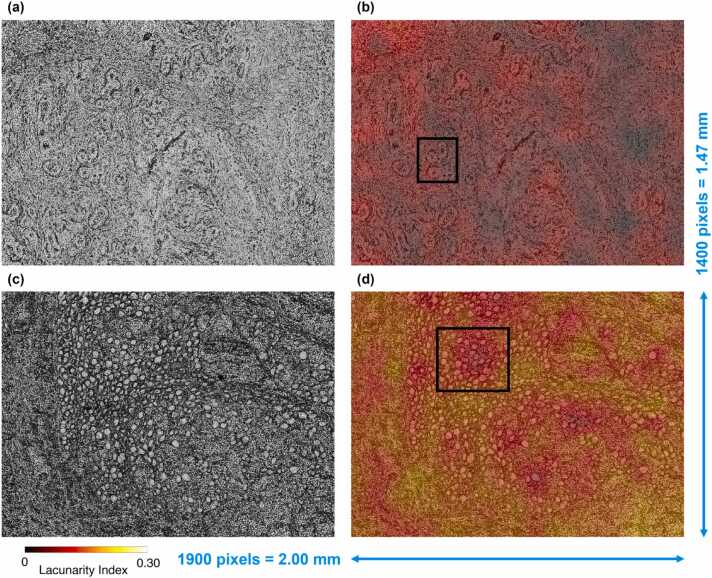
Visual inspection of the fibroadenoma (a,b) and breast cancer (c,d) tissue slides corresponding to the wrapped FPM maps in Fig. 3(a,b) and Fig. 3(c,d), respectively. (a,c) Low-resolution bright-field map. (b,d) Lacunarity heat maps of Supplementary Figs. S2(a,b), down-sampled and overlapped to the low-resolution bright-field maps in (a,c), respectively. Boxes in (b,d) highlight glandular structures.

In the proposed study, the lacunarity index characterizes the hole maps obtained from the wrapped FPM maps, as shown in Fig. 3. In particular, for each patch in Fig. 3(b,d), we calculated a lacunarity index, as displayed in Supplementary Figs. S2(a,b), respectively. In Supplementary Fig. S2, each 500 × 500 patch takes a homogeneous value, that is the corresponding lacunarity index. Hence, images in Supplementary Fig. S2 can be defined as the lacunarity heat maps. As the wrapped FPM maps have a high density of phase jumps (see Fig. 3(a,c)), it is difficult to correlate them to a specific biological structure by means of a visual inspection. Instead, for the sole purpose of a visual analysis performed by the pathologist, the low-resolution bright-field maps (1400 ×1900 square pixels) can be exploited, as displayed in Fig. 6(a,c), corresponding to the wrapped FPM maps in Fig. 3(a,c), respectively. To help the visual inspection and analysis by the pathologists, the lacunarity heat maps can be exploited. Again, lacunarity is calculated over the wrapped phase-contrast map. In particular, the 7000 × 9500 high-resolution lacunarity heat maps displayed in Supplementary Figs. S2(a,b) are resized to 1400 × 1900 square pixels in order to fit the size of the low-resolution bright-field map shown in Fig. 6(a,c), and to overlap them as in Fig. 6(b,d), respectively.

It is worth noting that the fibroadenoma tissue slide exhibits lower lacunarity indices than a breast cancer tissue slide, as respectively shown in Fig. 6(b,d). According to the definition of lacunarity index [52], this means that the fibroadenoma tissue slide is more lacunar than the breast cancer tissue slide. This property can be related to the different inner structures forming the two kinds of tissues, that can be seen in the low-resolution bright-field maps in Fig. 6(a,c). Small and rather spaced ductal-derived glandular structures are observed, in a fibrous stroma in Fig. 6(a,b), and the glandular density appears reduced. The heatmap in Fig. 6(b) highlights glandular spaced structures (see the black box). In Fig. 6(c,d), numerous ductal-derived glandular elements are observed tightly packed and without evident stroma. Glandular density appears high. Remarkably, the heatmap in Fig. 6(d) highlights numerous glandular structures bundled together (see the black box).

## 4 Discussion

Extraction of features from stained histology slides obtained through WSI has recently emerged as a pivotal technique in pathomic studies. The primary objective is to quantitatively characterize cells and tissues derived from examined samples. Notably, some pathomic studies delve into the fractal dimension analysis to extract features to analyse WSIs of various cancer types. For instance, Lee et al. developed a computer-aided technique for the automated grading of prostatic carcinoma leveraging the application of fractal dimension for analysing pathological image texture of prostatic carcinoma WSIs [74]. The extracted fractal dimension-based features were able to classify pathological prostate images into four classes within the Gleason grading system [74]. Furthermore, Da Silva et al. embraced fractal dimension analysis as a computationally accessible approach to enhance the histopathological diagnosis of breast cancer. Their investigation revealed that fractal dimension-based features extracted from stained WSI demonstrated remarkable capabilities in distinguishing breast carcinomas from normal tissue and benign breast alterations. These findings underscore the significance of fractal dimension analysis as a powerful tool in advancing our understanding and diagnostic capabilities in histopathological studies [75].

Here, it is important to address some critical considerations. The proposed histopathological analysis of breast cancer can be critical due to its intrinsic complexity. Indeed, cell density and morphology alone could not be sufficient for the differential diagnosis between a malignant and a benign lesion, as special cases such as florid adenosis or sclerosing adenosis could create outputs that may be associated with false-positive subjects. In addition, there are cases such as tubular breast carcinoma in which the tissue is well differentiated and only a detailed diagnostic study can make a correct diagnosis [76].

More in general, this paper is intended to show a proof-of-concept study of the applicability of FPM and the fractal analysis to the identification of fibroadenoma and breast cancer from paraffined unstained tissue slides. A future study involving a larger number of patients should follow this work in order to test the method on a clinically relevant sample of patients. Although the number of patients is limited to 10, the classifier operates in the first stage on single patches of single images taken from a set of measurements from each patient’s slide. Hence, in the initial stage, each image portion is classified independently, without taking into account the context of the entire image to which it belongs. The highly accurate classification performance achieved here is encouraging, prompting the expansion of the dataset to include a broader range of patients in future studies.

## 5 Conclusions

Digital pathology analysis of breast tissue slides is widespread and can furnish a valuable help to guide pathologists called to judge heterogeneous morphologies.

Here we established a novel analysis framework that allowed us to analyze directly the paraffined unstained breast slides. Avoiding these steps has to positive effect of getting rid of ambiguities that can be provoked by the paraffin removal and staining processes, which frequently arise in conventional breast cancer diagnosis. We used two multi-scale methodologies for image acquisition and analysis, respectively. Fractal biomarkers, in particular the lacunarity index, well describe the FPM phase-contrast maps and allow classifying data from the single image portion level up to the patient level. The analysis conducted in this study revealed that utilizing fractal descriptors on FPM maps enables the classifier to differentiate between image patches displaying the breast cancer phenotype and those displaying the fibroadenoma phenotype. Specifically, we observed a significant classification accuracy at the patch level (84.8% in testing), indicating the viability of implementing the subsequent step of classification refinement, which involves max-voting at both the image and patient levels.

In summary, the main contributions in this work are as follows:

1. We proposed an automatic method for classification of breast cancer and fibroadenoma based on the novel FPM technique applied to unstained tissue slides;
2. We introduced the fractal patterns analysis of wrapped FPM phase-contrast maps;
3. Histopathological image recognition, patch and patient classification are demonstrated by avoiding the removal of the paraffin layer;
4. Max-voting among different portions of the same image is demonstrated to enforce image classification, as discussed above. Besides, max-voting among different images from the same patient’s slide allowed very accurate classification. In particular, a 100% accuracy was obtained among the validation tissue slides of 4 patients and the test tissue slides of 4 patients after training a ML model with the tissue slides of other 2 patients. The robustness of this method would allow to judge even using a reduced set of FPM images for the same patient.
5. The most important fractal parameter, i.e. the lacunarity index, can serve to create guide maps for pathologists.

We believe that our approach is highly innovative and potentially usable in the future to support the pathologist's activities. In principle, the proposed strategy could be extended to other types of tissues. Therefore, next studies will be focused on handling the borderline and more challenging cases mentioned above. Generalization to other types of tissues and pathologies will be tested.

## Ethics approval and consent to participate

The study was approved by the Ethics Committee of IRCCS Pascale (Naples, Italy) with reference number 3/19 approved on 29 May 2019. All methods were performed in compliance with standard operating procedures and in accordance with the Declaration of Helsinki and each patient participated in the study by signing written informed consent.

## Author statement

As corresponding author of the manuscript “Classifying breast cancer and fibroadenoma tissue biopsies from paraffined stain-free slides by fractal biomarkers in Fourier Ptychographic Microscopy”, on behalf of all co-authors I hereby declare that this manuscript is original, has not been published before and is not currently being considered for publication elsewhere. Moreover, I confirm that the manuscript has been read and approved by all named authors and that there are no other persons who satisfied the criteria for authorship but are not listed. I further confirm that the order of authors listed in the manuscript has been approved by all of us.

## CRediT authorship contribution statement

**Giovanni Smaldone:** Writing – review & editing. **Massimiliano D’Aiuto:** Resources, Validation. **Gennaro Mossetti:** Resources, Validation. **Marco Salvatore:** Conceptualization, Funding acquisition, Project administration, Supervision. **Pietro Ferraro:** Conceptualization, Funding acquisition, Project administration, Supervision, Writing – original draft. **Vittorio Bianco:** Conceptualization, Investigation, Visualization, Writing – original draft, Writing – review & editing. **Marika Valentino:** Investigation, Visualization, Writing – original draft. **Daniele Pirone:** Conceptualization, Formal analysis, Visualization, Writing – original draft, Writing – review & editing. **Lisa Miccio:** Methodology. **Pasquale Memmolo:** Software. **Valentina Brancato:** Writing – review & editing. **Luigi Coppola:** Writing – review & editing.

## Declaration of Competing Interest

The authors have no conflict of interests to declare.

## Data Availability

Data used in this study are available from the corresponding author upon reasonable request.
