# Supplementary material for: Classifying breast cancer and fibroadenoma tissue biopsies from paraffined stain-free slides by fractal biomarkers in Fourier Ptychographic Microscopy
Source: Comput Struct Biotechnol J. 2024 Mar 24;24:225–36. doi: 10.1016/j.csbj.2024.03.019 (PMC10990711; doi:10.1016/j.csbj.2024.03.019)
Supplement: Supplementary file 1 — Supplementary material [file mmc1.pdf]

Supplementary Material of

Classifying breast cancer and fibroadenoma tissue biopsies from paraffined stain-free slides by fractal biomarkers in Fourier Ptychographic Microscopy

Vittorio Bianco,[a] Marika Valentino,[a,b] Daniele Pirone, [a,*] Lisa Miccio,[a] Pasquale Memmolo,[a] Valentina Brancato,[c] Luigi Coppola,[c] Giovanni Smaldone,[c] Massimiliano D'Aiuto,[d] Gennaro Mossetti,[e] Marco Salvatore,[c] and Pietro Ferraro[a]

[a] CNR-ISASI, Institute of Applied Sciences and Intelligent Systems "E. Caianiello", Via Campi Flegrei 34, 80078 Pozzuoli, Napoli, Italy.
[b] DIETI, Department of Electrical Engineering and Information Technologies, University of Naples "Federico II", via Claudio 21, 80125 Napoli, Italy.
[c] IRCCS SYNLAB SDN, Via E. Gianturco 113, Napoli, 80143, Italy.
[d] Clinica Villa Fiorita, Via Filippo Saporito 24, 81031 Aversa, Caserta, Italy.
[e] Pathological Anatomy Service, Casa di Cura Maria Rosaria, Via Colle San Bartolomeo 50, 80045 Pompei, Napoli, Italy.

*daniele.pirone@isasi.cnr.it

S1 Fractal features

Here we describe the set of 15 fractal parameters listed in Section 2.3 and used to characterize and classify the single FPM patches (500×500) belonging to fibroadenoma and breast cancer tissue slides. As the numerical methods usually employed to compute fractal parameters work with powers of 2, each patch is zero-padded up to 512×512 square pixels. Let ψ be the wrapped FPM map of a single patch (see the insets in Figs. 3(a,c)). Let Γ_ψ be the hole FPM map obtained by zero-thresholding the corresponding wrapped FPM map ψ (see the insets in Figs. 3(b,d)). Let S_ψ be the support FPM map, i.e. a map of all ones having the same size of ψ. Hence, the wrapped FPM map ψ, the hole FPM map Γ_ψ, and the support FPM map S_ψ have $l = 512$ pixels per side and are exploited to compute fractal features [52].

Fractal dimension is computed through the box-counting method [S1], which is based on the Minkowski–Bouligand definition [S2]. An image of size l can be covered by ε^2 non-overlapping boxes of size $r = 1,2,4,8,\dots,l$, where $\varepsilon = l/r$ is defined as scale factor. By fixing a certain scale factor ε, $n(\varepsilon)$ is computed as the number of non-overlapping $r \times r$ boxes containing at least a non-zero element in the hole FPM map Γ_ψ. According to the Minkowski–Bouligand definition [S2], the fractal dimension D can be written as

$$D = \lim_{1/\varepsilon \to 0} \frac{\log_2 n(\varepsilon)}{\log_2 \varepsilon}, \qquad \text{(S1)}$$

i.e. it is numerically computed as the slope of the linear fitting to the log-log plot of $n(\varepsilon)$ measurements [52]. Hence, unlike the topological dimension (i.e., 0 for a point, 1 for a line, 2 for a plane object and 3 for a solid object), the fractal dimension can also take non-integer values.

Lacunarity allows measuring the distribution of the hole sizes in an image and it is usually calculated through the gliding box algorithm [S3]. In fact, unlike fractal dimension, to compute lacunarity, gliding boxes instead of non-overlapping boxes are used to scan the map at different scales $\varepsilon = 1,2,4,8,\dots l$. Let $A(\varepsilon)$ and $B(\varepsilon)$ be the 2D convolutions of respectively $1 - \Gamma_\psi$ and S_ψ with $H(\varepsilon)$, that is a $r \times r$ matrix with all ones. The probability distribution related to $A(\varepsilon)$ is defined as $m(a, \varepsilon)$,

with $a = 1,2,\dots,r^2$, and is obtained by dividing the frequency distribution by the number of non-zero elements in $B(\varepsilon)$. The first and second moments of $m(a,\varepsilon)$ are defined as $p_1(\varepsilon)$ and $p_2(\varepsilon)$, respectively. Then, the lacunarity function is computed as [S3]

$$\Lambda(\varepsilon) = \frac{p_2(\varepsilon)}{p_1^2(\varepsilon)}. \tag{S2}$$

Finally, the lacunarity index is defined as the exponential coefficient $L = b_2$ of the exponential fitting $y = b_0 + b_1 e^{b_2 x}$ to the curve $\Lambda(\log_2(\varepsilon))$ [52]. It is worth noting that a low lacunarity index L is associated with a big lacunarity.

The fill ratio is the ratio between the number of non-zero elements within the hole FPM map Γ_ψ and the support FPM map S_ψ.

Regularity measures how uniformly distributed are the zero and non-zero values with respect to the hole FPM map Γ_ψ [S3]. Let P be a generic point in the support FPM map S_ψ (corresponding to Z if zero in the hole FPM map Γ_ψ, otherwise $\bar{Z}$), which is centered in C. The elements of vectors $\underline{d}_{x,P}$, $\underline{d}_{x,Z}$ and $\underline{d}_{x,\bar{Z}}$ are the differences between the x-coordinates of point C and points P, Z and $\bar{Z}$, respectively. The elements of vectors $\underline{d}_{y,P}$, $\underline{d}_{y,Z}$ and $\underline{d}_{y,\bar{Z}}$ are the differences between the y-coordinates of point C and points P, Z and $\bar{Z}$. The Pearson Correlation Coefficients [S4] $R_{x,Z}$, $R_{y,Z}$, $R_{x,\bar{Z}}$ and $R_{y,\bar{Z}}$ are then computed between the histograms (normalized to their maxima) of vectors $\underline{d}_{x,Z}$ and $\underline{d}_{x,P}$, $\underline{d}_{y,Z}$ and $\underline{d}_{y,P}$, $\underline{d}_{x,\bar{Z}}$ and $\underline{d}_{x,P}$, $\underline{d}_{y,\bar{Z}}$ and $\underline{d}_{y,P}$, respectively. The regularity index is finally calculated as the absolute value of the mean among these four correlation coefficients [52]. If the zero elements Z and the non-zero elements $\bar{Z}$ in the hole FPM Γ_ψ have the same spatial distribution of the non-zero elements in the support FPM map S_ψ, the maximum value $R = 1$ is obtained.

Moreover, for both curves $n(\varepsilon)$ and $\Lambda(\varepsilon)$,

$$\begin{aligned} dn\,(\varepsilon) &= \frac{\nabla \log_2 n(\varepsilon)}{\nabla \log_2 \varepsilon} \\ d\Lambda\,(\varepsilon) &= \frac{\nabla \Lambda(\varepsilon)}{\nabla \varepsilon} \end{aligned} \tag{S3}$$

are calculated by considering ∇ as the gradient operator. The fractal dimension contrast C_D and the lacunarity contrast C_L are defined as the ratio between the standard deviations and the average values of $dn\,(\varepsilon)$ and $d\Lambda\,(\varepsilon)$, respectively [52].

In the case of a non fractal object such as an $l \times l$ image with all ones, $dn\,(\varepsilon) = 2$ for any $\varepsilon = 1,2,4,8,\dots l$. In the case of a non fractal object such as a $l \times l$ image with all zeros, $d\Lambda(\varepsilon) = 0$ for any $\varepsilon = 1,2,4,8,\dots l$. The fractal dimension RMSE E_D is defined as the root mean square error (RMSE) between $dn\,(\varepsilon)$ and $dn\,(\varepsilon) = 2$, while the lacunarity RMSE E_L is defined as the RMSE between $d\Lambda(\varepsilon)$ and $d\Lambda(\varepsilon) = 0$ [52].

The vertex density V is the ratio between the number of corners of the hole FPM map Γ_ψ and the number of non-zero values of the support FPM map S_ψ [52].

Let the vertex FPM map V_ψ be a new $l \times l$ image obtained from the the support FPM map S_ψ by setting to 0 pixels identified as corners in the hole FPM map Γ_ψ. By using the the vertex FPM map V_ψ instead of the hole FPM map Γ_ψ, the vertex lacunarity index L_V, vertex regularity index R_V, vertex lacunarity contrast C_{L_V}, and vertex lacunarity RMSE E_{L_V} are computed [52].

Finally, the standard deviation and the entropy are calculated directly from the wrapped FPM map ψ.

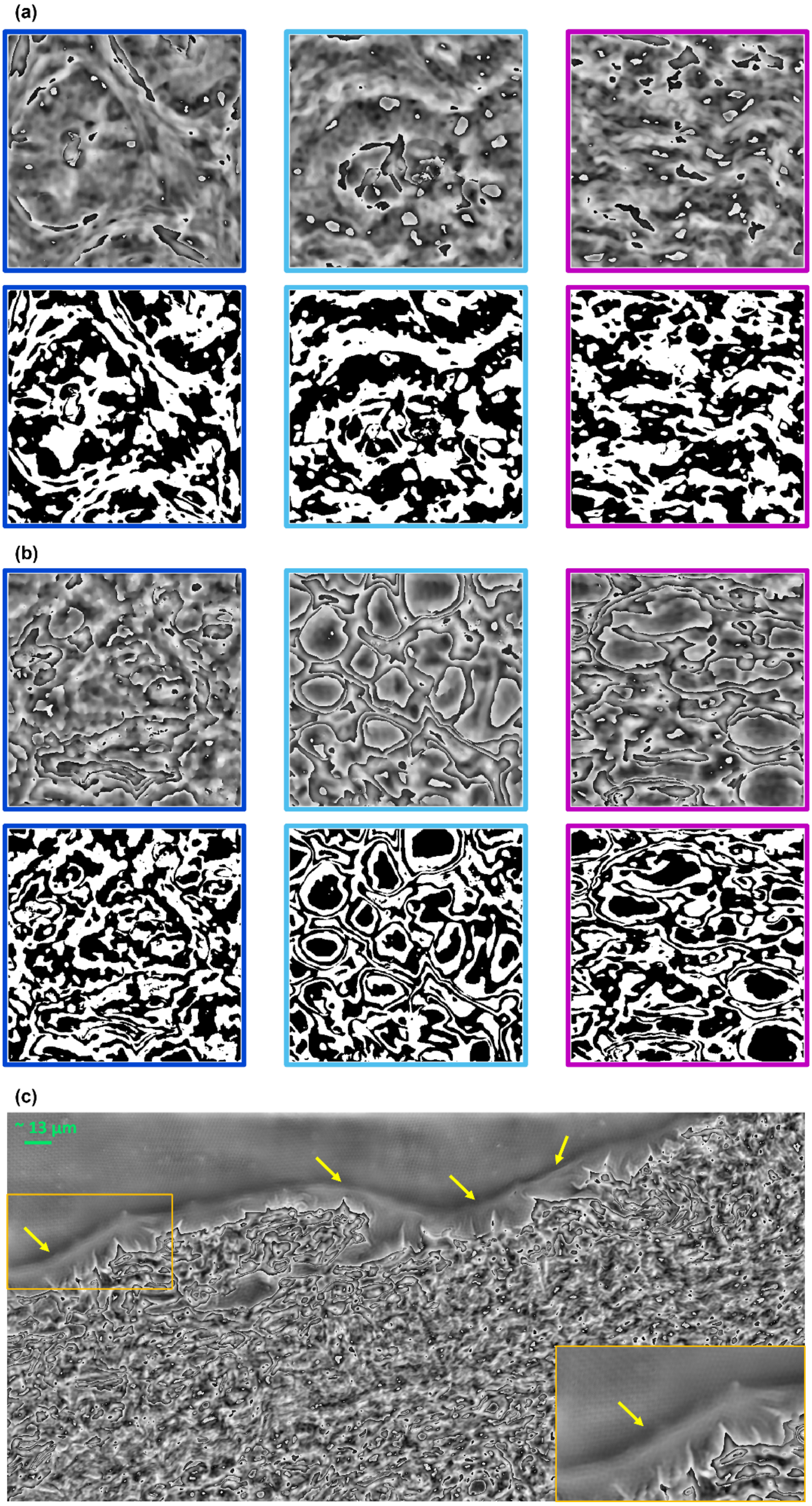

Figure S1. Details about the FPM images of breast tissue slides. (a) Insets of the fibroadenoma tissue slide in (top) Fig. 3(a) and (bottom) Fig. 3(b). **(b)** Insets of the breast cancer tissue slide in (top) Fig. 3(c) and (bottom) Fig. 3(d). **(c)** Portion of stain-free paraffined fibroadenoma tissue slide. The yellow arrows indicate the areas where the effect of the sole paraffin layer can be appreciated.

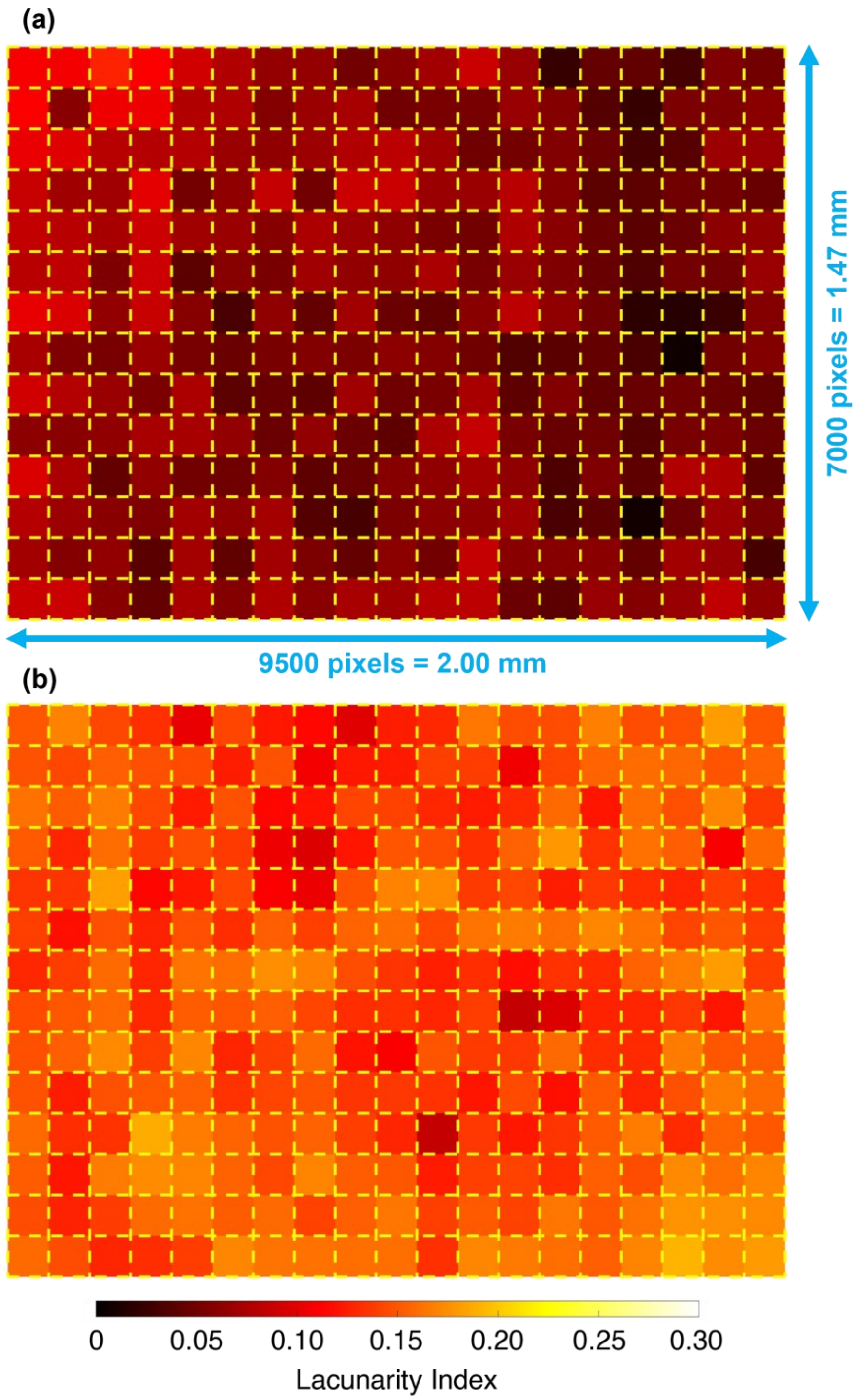

Figure S2. Lacunarity heat map made of the lacunarity index values related to each 500x500 patch dividing the full 7000x9500 FPM FOV. (a) Fibroadenoma tissue slide corresponding to that in Figs. 2(a,b). **(b)** Breast cancer tissue slide corresponding to that in Figs. 2(c,d).

References

S1. Moisy, F. (2024). Boxcount, MATLAB Central File Exchange. Retrieved 05/03/2024. (https://www.mathworks.com/matlabcentral/fileexchange/13063-boxcount).

S2. Bouligand, G. Sur la notion d'ordre de mesure d'un ensemble plan. Bull. Sci. Math. 1929, 2, 185-192.

S3. Plotnick, R. E.; Gardner, R. H.; O'Neill, R. V. Lacunarity indices as measures of landscape texture. Landsc. Ecol. 1993, 8, 201-211.

S4. Folks, J. L.; Chhikara, R. S. The inverse Gaussian distribution and its statistical applications-a review. J. R. Stat. Soc. Series B Stat. Methodol. 1978, 40, 263–289.
